# Dataset of pesticides, pharmaceuticals and personal care products occurrence in wetlands of Saudi Arabia

**DOI:** 10.1016/j.dib.2020.105776

**Published:** 2020-05-29

**Authors:** Rodrigo Álvarez-Ruiz, Yolanda Picó, Ahmed H. Alfarhan, Mohamed A. El-Sheikh, Hamad O. Alshahrani, Damià Barceló

**Affiliations:** a Environmental and Food Safety Research Group (SAMA-UV), Desertification Research Centre CIDE (CSIC-UV-GV), Moncada-Naquera Road Km 4.5, 46113 Moncada, Spain; b Department of Botany and Microbiology, College of Science, King Saud University, P.O. Box 2455, Riyadh 11451, Saudi Arabia; c Water and Soil Quality Research Group, Department of Environmental Chemistry, IDAEA-CSIC, Barcelona, Spain

**Keywords:** Emerging pollutants, wetland, water discharge, water, sediment, soil, plants

## Abstract

The data set presents the occurrence of 59 currently used pesticides (CUPs) and 33 pharmaceuticals and personal care products (PPCPs), from wetland areas, in Saudi Arabia, impacted by wastewater discharge. Wetlands are valuable ecosystems, but are very fragile and easily affected by anthropogenic pressure [1], [2], [3], [4], [5], [6]. The occurrence of organic contaminants provides understanding about their fate and possible risk for humans and environment. Up to our knowledge, this is the first report on the occurrence of the mentioned organic pollutants in shallow lakes in Saudi Arabia, and the first time these compounds are analyzed in wild flora. Samples of water, sediment, soil and plants were extracted via ultrasound assisted extraction (UAE) and solid phase extraction (SPE). The compounds determination was performed using ultra-high-performance liquid chromatography tandem mass spectrometry (HPLC-MS/MS). Interpretation and discussion of the present dataset can be found in the article entitled “*Pharmaceuticals, pesticides, personal care products and microplastics contamination assessment of Al-Hassa irrigation network (Saudi Arabia) and its shallow lakes”*[1].

Specifications table

**Table:** 

Subject	Pollution
Specific subject area	Organic pollutants occurrence and fate in wetland areas affected by wastewater discharge
Type of data	Table
How data were acquired	The data were acquired via liquid chromatography-mass spectrometry. The instruments were a 1260 Infinity Ultra-High-Performance Liquid Chromatograph (UHPLC) combined with an Agilent 6410 Triple Quadrupole (QqQ) Mass Spectrometer (MS/MS), with an electrospray ionizer (ESI) (Agilent Technologies, Santa Clara, CA, USA). Data were processed using a MassHunter Workstatin Software for qualitative and quantitative analysis (GL Sciences, Tokyo, Japan).
Data format	Raw, Analyzed, Filtered, Tables and graphs. The results presented are average of triplicate sample analysis.
Parameters for data collection	The mobile phases were methanol and water: 10mM ammonium formate for pesticides, 0.1 % formic acid for positive ionization PPCPs and 2.5mM NH_4_F for negative ionization PPCPs. The rest of the parameters are specified in the literature [1].
Description of data collection	Concentration of 59 CUPs and 33 PPCPs, were obtained analysing the extracts of environmental samples (water, sediment, soil and plants) collected in the eastern region of Saudi Arabia. The extraction procedures are detailed in the experimental design, materials, and methods section.
Data source location	Institution: Environmental and Food Safety Research Group (SAMA-UV), Desertification Research Centre CIDE (CSIC-UV-GV) City/Town/Region: Moncada, Community of Valencia Country: Spain Latitude and longitude (and GPS coordinates) for collected samples: Al-Asfar Site 1 (25° 24′3.04" N 49°43′36.20" E), Al-Asfar Site 2 (25° 30′43.07" N 49°45′17,57" E), Al-Asfar Site 3 (25° 30′48.30" N 49°47`6.33" E), Al-Asfar site 4 (25° 30′45.36" N 49°49′58.06" E), Al-Asfar site 5 (25° 34′41.71" N 49°52′18.55" E), Al-Hubail site 1 (25°36′38.30" N 49°38′48.05" E), Al-Hubail site 2 (25°39′42.30" N 49°39′15.52" E), Al-Hubail site 3 (25°40′41.24" N 49°40′1.69" E), Al-Hubail site 4 (25°40′39.01" N 49°40′58.99" E), Al-Hubail site 5 (25°41′42.12" N 49°43′12.49" E).
Data accessibility	Data are available with the article
Related research article	Picó, Y., Alvarez-Ruiz R., Alfarhan A. H., El-Sheikh M. A., Alshahrani H. O., Damià Barceló D., *Pharmaceuticals, pesticides, personal care products and microplastics contamination assessment of Al-Hassa irrigation network (Saudi Arabia) and its shallow lakes,* Science of The Total Environment, 2020. 701: p. 135021, https://doi.org/10.1016/j.scitotenv.2019.135021

## Value of the data

• The analysis of occurrence of organic pollutants in the environment is needed to assess their risk and fate.
• Concentration values can be used by other researchers and local authorities.
• The occurrence can be useful for supporting further research of the risk and fate of organic compounds, restoration policies and contaminant elimination measures, among others.
• The data of every sampling point provides a better understanding in the distribution of the organic compounds
• The tables offer a comprehensive overview of the occurrence of a wide range of pharmaceuticals and PCPs in water, sediment, soil and plants of a very little studied area.
• These data can be a useful contribution to prioritisation exercises as well as to establish environmental quality standards.

## Data Description

The following dataset shows 4 tables with the occurrence of CUPs and PPCPs in the different environmental matrices. For both shallow lakes, Al-Asfar and Al-Hubail, the sites 3, 4 and 5 were located in the shallow lake, while site 1 was located in irrigation channels, which provide wastewater (from farms, factories and/or domestic sewage) to each lake. Site 2 was located between the end of the irrigation channels and the mouth of each lake. Detailed information of each sampling site is provided in the related article [1]. Table 1 shows the occurrence of CUPS and PPCPs in water samples, while tables 2, 3 and 4 show the occurrence in sediments, soil and plants (wild flora *Phragmites australis*) respectively. In order to make the table easier to understand the data has been filtered, eliminating in each table, those compounds that were not detected in the sampling sites. A detailed list of the analyzed compounds is provided in the related article [1]. Furthermore, the CUPs acetochlor, acrinathrin, alachlor, atrazine, atrazine-deethyl, atrazine-deisopropyl, azinphos-ethyl, azinphos-methyl, buprofezin, carbofuran, chlotianidin, coumaphos, diclofenthion, dimethoate, diuron, 2,4-dimethylaniline (DMA), 2,4-dimethylphenylformamide (DMF), 2,4-dimethylphenyl-N′-methylformamidine (DMPF), ethion, etofenprox, fenthion, fenthion sulfoxide, fipronil, flumethrin, hexythiazox, malathion, methiocarb, metolachlor, molinate, omethoate, parathion-ethyl, parathion-methyl, propanil, propazine, pyriproxyphen, simazine, spinosyn A, spinosyn C, spinosyn D, terbumeton, terbumeton-deethyl, terbutryn and tolclophos-methyl and the PPCPs allopurinol, amoxicillin, chloramphenicol, furosemide, indomethacin, norfloxacin and thiamphenicol were not detected in the samples. In addition, the tables also show the total accumulated contamination for each contaminant and matrix, which provides and insight of the overall presence (and use) of each compound in the area.

**Table 1:** Occurrence of CUPs and PPCPs in water of the study area.

	Concentration in water (ng L^−1^)										
	Al-Asfar					Al-Hubail					
**CUPs**	Site 1	Site 2	Site 3	Site 4	Site 5	Site 1	Site 2	Site 3	Site 4	Site 5	**Total per compound**
Acetamiprid	10.10	12.24	7.17	-	-	-	-	-	-	-	30.01
Bifenthrin	-	-	0.32	0.76	0.19	40.82	45.28	-	-	-	87.37
Carbendazim	40.71	25.34	38.52	2.53	4.63	153.73	192.91	141.80	15.29	25.54	641.00
Carbofuran-3-hydroxy	102.09	-	4.93	-	-	-	-	-	-	-	107.02
Chlorfenvinphos	-	11.22	-	7.99	-	-	-	-	5.43	10.93	36.02
Chlorpyrifos	-	-	-	1.11	1.94	-	-	-	23.65	24.33	51.04
Cyhalothrin	-	-	-	5.73	-	-	-	63.87	-	-	69.59
Diazinon	1016.04	131.98	151.95	1.12	1.18	37.15	40.08	42.10	-	-	1421.58
Fluvalinate	-	-	1.31	-	-	-	-	-	-	-	1.31
Imazalil	18.32	8.93	6.98	-	-	-	-	-	-	-	34.22
Imidacloprid	94.03	58.80	42.88	4.63	-	445.00	103.33	-	-	-	748.65
Isoproturon	-	-	-	-	-	-	-	67.42	-	-	67.42
Tebuconazole	-	-	7.81	-	-	-	-	-	-	-	7.81
Terbuthylazine-2-hydroxy	-	-	3.11	10.27	11.57	-	-	-	-	-	24.94
Thiabendazole	22.42	15.05	18.59	-	-	-	10.06	-	9.70	-	75.81
Thiametoxan	-	-	10.82	-	-	-	-	-	-	-	10.82
**PPCPs**											
Alprazolam	-	343.65	-	287.00	286.00	329.05	317.16	318.65	382.48	389.47	2653.46
Atenolol	-	326.97	219.88	75.05	100.02	60.57	117.67	145.72	82.58	116.06	1244.52
Atorvastatin	474.69	360.36	249.95	203.25	198.00	272.57	23.55	191.79	238.27	211.95	2424.38
Bisphenol A	337.50	484.86	195.05	-	105.09	258.19	185.20	95.82	100.25	140.25	1902.21
Buthylparaben	60.20	56.80	57.06	65.22	-	58.02	58.02	-	-	-	355.32
Caffeine	20663.48	11425.67	5217.00	294.54	269.74	1906.00	2721.26	1372.83	390.83	230.31	44491.66
Clofibric acid	-	-	-	-	-	-	1.52	-	-	-	1.52
Codeine	22.46	-	-	-	-	-	-	-	-	-	22.46
Diclofenac	1390.00	937.40	584.45	10.25	10.25	50.02	45.29	-	-	-	3027.66
Ethylparaben	6.25	-	-	-	-	-	1.52	-	-	-	7.77
Etoricoxib	376.72	462.60	463.11	433.96	433.08	433.55	382.89	473.96	445.61	456.97	4362.45
Ibuprofen	2407.00	685.65	1312.25	72.80	106.25	638.25	3.73	102.05	-	-	5327.98
Lorazepam	480.44	501.25	463.47	496.34	442.78	422.00	415.16	472.50	506.86	472.87	4673.67
Metformin	267.01	173.85	91.86	10.39	9.26	32.61	33.44	152.92	1.98	3.16	776.48
Methylparaben	27.40	22.50	-	10.05	5.25	2.54	2.05	-	-	-	69.79
Naproxen	142.95	-	-	-	-	-	15.98	-	-	-	158.93
Ofloxacin	610.58	393.85	216.36	178.58	152.29	278.33	283.59	215.27	212.77	147.99	2689.61
Paracetamol	3069.06	546.74	196.92	154.83	147.79	175.71	721.98	163.18	109.28	105.06	5390.55
Propylparaben	12.54	-	-	-	-	-	1.25	-	-	-	13.79
Salicylic acid	129.20	120.30	84.45	76.75	5.25	92.23	104.85	45.00	45.00	62.01	765.04
Tramadol	301.17	314.15	346.85	305.25	310.24	313.25	326.74	353.46	324.28	289.89	3185.28
Triclocarban	32.00	18.25	15.09	5.22	-	16.29	16.29	-	-	-	103.14
Triclosan	33.52	21.45	10.08	-	-	25.59	25.59	-	-	-	116.23
Trimetroprim	586.25	236.42	-	-	-	-	-	-	-	-	822.67

-: not detected

**Table 2:** Occurrence of CUPs and PPCPs in sediment of the study area.

	Concentration in Sediment (ng g^−1^)										
	Al-Asfar					Al-Hubail					
**CUPs**	Site 1	Site 2	Site 3	Site 4	Site 5	Site 1	Site 2	Site 3	Site 4	Site 5	**Total per compound**
Atrazine	-	-	-	-	-	0.01	-	-	-	-	0.01
Chlorfenvinphos	1.25	0.56	1.16	-	0.70	0.82	1.25	0.49	1.06	0.54	7.83
Chlorpyrifos	0.21	0.33	0.23	-	-	0.20	0.21	0.28	0.24	0.20	1.90
Cyhalothrin	-	-	-	-	-	0.19	-	-	-	-	0.19
Diazinon	-	-	-	-	-	-	0.03	-	-	-	0.03
Imazalil	0.40	-	-	-	-	-	-	-	-	-	0.40
Imidacloprid	0.40	-	0.86	2.16	-	0.99	0.40	0.37	-	9.09	14.28
Terbuthylazine	-	0.27	0.32	-	-	0.83	0.06	-	0.17	-	1.65
Terbuthylazine-deethyl	-	0.05	-	-	-	-	-	-	-	-	0.05
**PPCPs**											
Alprazolam	79.19	82.25	-	75.32	77.80	82.45	80.08	87.00	-	-	564.09
Atenolol	5.78	-	-	-	2.50	13.51	7.08	11.35	11.35	7.10	58.67
Atorvastatin	84.49	68.72	14.00	56.21	21.00	35.08	49.24	28.96	47.00	32.00	436.70
Bisphenol A	65.35	35.33	-	88.41	12.43	90.85	24.86	3.22	9.85	12.87	343.17
Buthylparaben	-	11.53	-	-	11.36	-	-	-	-	-	22.89
Caffeine	13.32	75.96	7.07	11.58	25.53	53.02	54.85	64.02	64.00	42.00	411.35
Diclofenac	-	4.90	0.60	-	21.73	-	1.86	-	-	-	29.09
Etoricoxib	8.22	63.61	0.70	2.37	6.50	63.95	51.43	49.39	9.90	6.00	262.07
Ibuprofen	-	-	-	-	23.97	-	-	-	-	-	23.97
Lorazepam	126.46	120.49	116.00	123.04	115.00	109.68	100.58	118.00	111	120.00	1160.25
Metformin	-	-	-	0.19	-	0.27	0.10	0.32	0.60	0.28	1.76
Ofloxacin	-	-	-	-	-	17.16	-	-	-	-	17.16
Paracetamol	15.35	24.98	14.78	15.98	15.19	12.51	11.55	21.89	17.10	17.41	166.74
Salicylic acid	15.33	6.44	4.83	7.23	-	17.69	11.20	6.62	11.20	12.07	92.61
Simvastatin	472.95	379.11	38.36	63.16	557.00	388.00	589.27	510.00	476.00	419.00	3892.85
Tramadol	69.82	107.11	11.30	34.62	80.52	86.13	76.90	92.08	68.00	52.00	678.48
Triclocarban	-	-	4.67	-	-	10.36	4.19	-	-	-	19.22

-: not detected

**Table 3:** Occurrence of CUPs and PPCPs in soil of the study area.

	Concentration in soil (ng g^−1^)										
	Al-Asfar					Al-Hubail					
**CUPs**	Site 1	Site 2	Site 3	Site 4	Site 5	Site 1	Site 2	Site 3	Site 4	Site 5	**Total per compound**
Carbendazim	-	-	-	-	-	-	0.04	-	-	-	0.04
Chlorfenvinphos	-	-	-	-	-	-	0.44	0.84	0.74	0.69	2.71
Chlorpyrifos	0.66	0.44	0.34	0.57	0.20	0.28	0.55	0.44	0.62	0.84	4.94
Fenitrothion	-	-	-	-	-	56.10	-	-	-	-	56.10
Imidacloprid	-	0.28	-	-	-	-	-	-	-	-	0.28
**PPCPs**											
Bisphenol A	18.07	32.23	19.47	3.87	22.77	15.76	45.25	19.55	19.79	8.75	205.51
Caffeine	2.89	12.49	25.44	3.32	1.74	5.15	4.55	2.36	4.39	3.32	65.65
Diclofenac	-	-	2.08	-	1.25	12.46	5.03	4.06	-	-	24.88
Ethylparaben	-	-	-	-	-	-	0.20	-	-	-	0.20
Ibuprofen	-	59.57	1.44	-	-	-	-	-	-	-	61.01
Metformin	-	-	-	-	-	0.28	0.67	-	-	-	0.95
Salicylic acid	18.03	9.45	13.23	6.68	6.17	76.07	9.44	9.38	10.77	11.20	170.42
Tramadol	-	-	1.76	-	-	-	-	-	-	-	1.76
Triclocarban	-	-	1.91	-	-	-	-	-	-	-	1.91
Triclosan	-	7.34	-	-	1.59	-	-	-	-	3.14	12.07

-: not detected

**Table 4:** Occurrence of CUPs and PPCPs in plants of the study area.

	Concentration in plants (ng g^−1^)										
	Al-Asfar					Al-Hubail					
**CUPs**	Site 1	Site 2	Site 3	Site 4	Site 5	Site 1	Site 2	Site 3	Site 4	Site 5	**Total per compound**
Carbendazim	-	-	-	0.34	0.35	-	-	-	-	-	0.69
Chlorfenvinphos	-	-	-	0.90	-	-	-	0.87	-	-	1.77
Chlorpyrifos	0.65	0.44	0.21	0.47	0.36	0.49	0.44	0.44	-	-	3.50
Diazinon	-	-	0.84	2.67	-	2.63	-	-	-	-	6.14
Fenthion sulfone	53.88	62.33	-	-	-	-	-	-	-	55.62	171.83
Prochloraz	-	-	-	0.49	-	0.41	-	-	-	-	0.9
Terbuthylazine-deethyl	-	-	-	-	-	-	1.28	-	-	-	1.28
**PPCPs**											
Atorvastatin	-	-	-	-	-	5.67	16.70	-	-	-	22.37
Bezafibrate	-	-	-	-	-	62.06	17.52	-	-	-	79.58
Bisphenol A	96.72	45.13	51.28	36.97	38.83	126.18	3.18	96.72	28.19	15.38	538.58
Caffeine	-	-	5.42	3.36	3.05	-	-	3.01	3.01	-	22.61
Diclofenac	-	-	-	-	-	-	-	16.04	-	-	16.04
Ibuprofen	-	-	-	-	-	135.16	-	-	-	-	135.16
Metformin	1.14	1.40	0.75	0.29	0.29	1.28	-	0.71	0.26	27.87	33.99
Methylparaben	59.97	79.52	32.08	11.07	614.34	95.48	27.56	144.25	124.78	-	1189.05
Naproxen	-	-	-	-	-	-	67.66	-	-	-	67.66
Ofloxacin	-	-	99.48	-	-	-	-	-	-	-	99.48
Paracetamol	28.34	10.40	-	-	-	-	-	-	-	-	38.74
Salicylic acid	1952.00	218.37	317.55	90.74	313.54	151.67	147.26	272.54	779.86	680.79	4924.32
Tramadol	-	1.16	-	-	-	-	-	-	-	-	1.16
Triclocarban	-	-	-	-	-	-	0.21	-	-	-	0.21

-: not detected

**Fig. 1:**
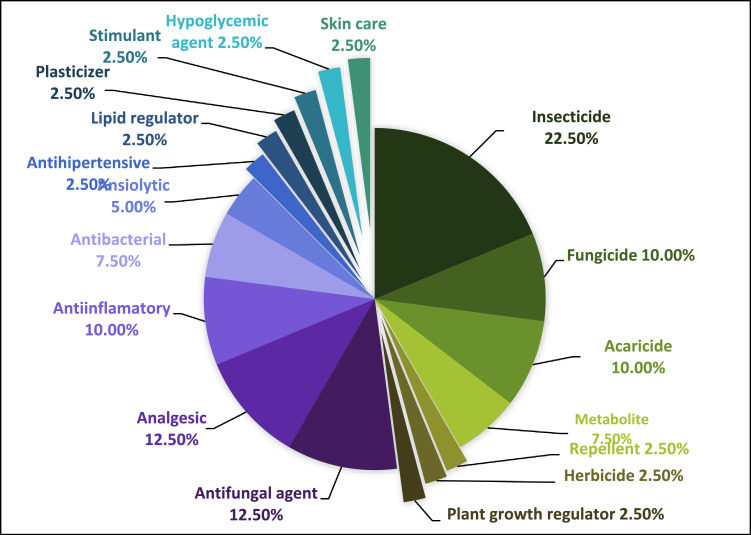
Actions of the 40 compounds detected in the water samples of the study area.

**Fig. 2:**
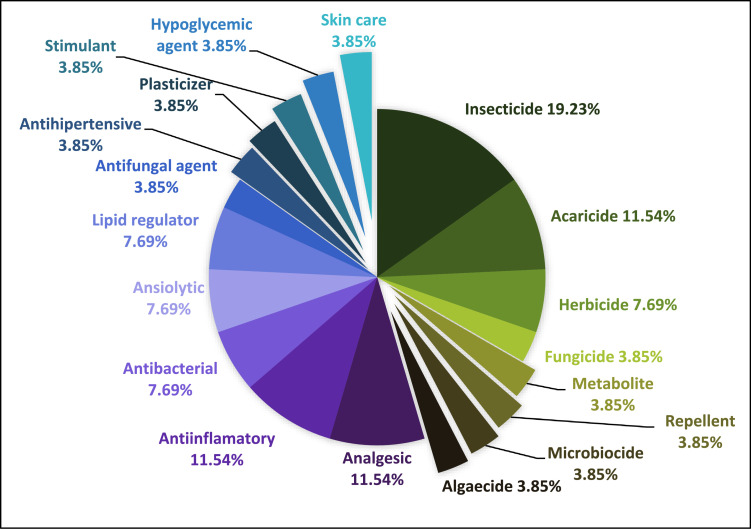
Actions of the 26 compounds detected in the sediment samples of the study area.

**Fig. 3:**
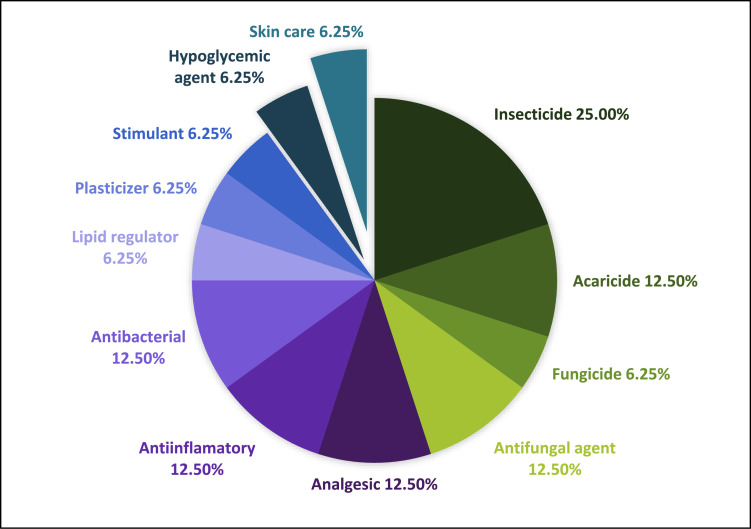
Actions of the 16 compounds detected in the soil samples of the study area.

**Fig. 4:**
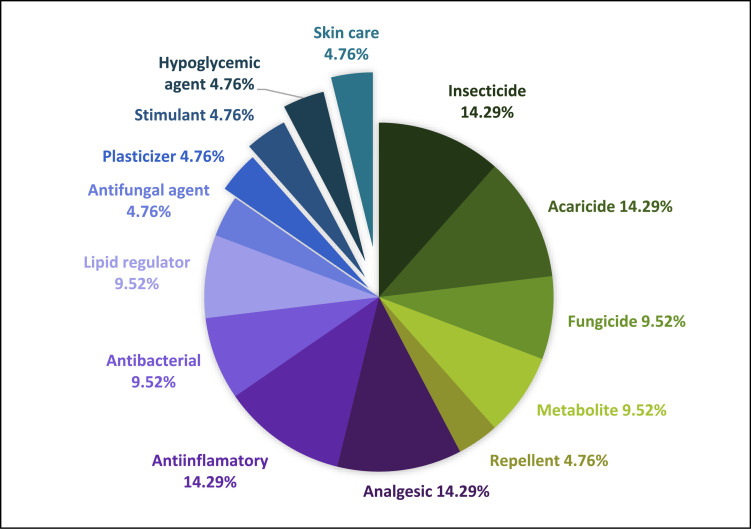
Actions of the 21 compounds detected in the plant samples of the study area.

In the figures are represented the different the actions of the compounds detected in the environmental matrices. Since there are compounds with more than one action, the sum of the percentages of each figure overcomes 100%. Figures 1, 2, Fig. 3, Fig. 4 show these actions of the compounds detected in water, sediment, soil and plants respectively. Information about the specific actions of each compound is provided in the related article [1]. These figures provide understanding about population requirements, regardless the compounds used to satisfy them.

## Experimental Design, Materials, and Methods

Once at the laboratory, surface water samples were filtered with glass microfiber filters (90 mm Ø) and stored at -20 °C until the analysis by solid-phase extraction (SPE) following a previously described method [2] and this information is also available in the related research article [1]. For the SPE Phenomenex Strata-X33u Polymeric Reversed Phase (200 mg/6 mL) cartridges (Phenomenex, Torrance, California, USA) and a vacuum manifold Supelco Visiprep 57030-U (Sigma-Aldrich, St. Louis, Missouri, USA) were used. The cartridges were conditioned with 6 mL of MeOH and 6 mL of Milli-Q water under vacuum at 400 mba h^−1^ Pa^−1^. Two-hundred and fifty mL of samples were measured in a volumetric flask, and spiked with the internal standard (IS) to obtain a final concentration in the vial of 20 ng mL^−1^. Then, each sample was passed through a cartridge at flow rate of 10 mL min^−1^ (wise drop). Then, the cartridges were washed with 6 mL of Milli-Q and dried for 15 min, both steps were performed under vacuum. The analytes were eluted on a 15 mL plastic Falcon tube with 6mL of MeOH and then 3 mL of MeOH-dichloromethane (DCM) solution (1:1, v/v) at gravity flow. Vacuum was just used at the beginning of the elution to break the superficial tension, and at the end, to collect the remaining drops of extract from the cartridges. Extracts were evaporate to dryness at 40°C, under a gentle stream of nitrogen, in a combined sample concentrator model SBHCONC/1 and heating plate model SBH130D/3 (Stuart®UK). The residue was redissolved in 1 mL of MilliQ water-MeOH (70:30, v/v), vortex for 1 min and sonicated for 1 min. Finally, each extract was stored in 2 mL amber vials with stoppers 99mm+Septum Sil/PTFE, (Análisis Vínicos S.L., Tomelloso, España), at -20°C until analysis.

Lyophilized sediment, soil and plant were sieved (2 mm Ø) and extracted by ultrasound assisted extraction (UAE) using the McIlvaine–EDTA method, followed by the same SPE clean-up procedure as used for water samples [3]. To perform the UAE McIlvaine-EDTA buffer was prepared mixing 100 mL of 0.1 M citric ac. solution, 62.5 mL of 0.2 M Na_2_HPO_4_ solution and 6.05 g of Na_2_-EDTA. Using MilliQ water as solvent. Then 1 g of sample was placed in a 50 mL Falcon plastic tube and spiked with the IS as described before. Then 5 mL of MeOH, 5 mL of MilliQ water and 5 mL of the MCIlvaine-EDTA buffer were added. The mix was vortex for 3 min, sonicated for 15 min and centrifuged for 6 min at 1811 rcf. The supernatant was collected in a 250 mL volumetric flask, filled with MilliQ water. Then the SPE was applied as described before.

The conditions used for the LC-MS/MS are exhaustively detailed in the related article, as well as the identification, characterization and main properties of the target analytes [1].

## Declaration of Competing Interest

The authors declare that they have no known competing financial interests or personal relationships, which have, or could be perceived to have, influenced the work reported in this article.
